# Building blocks for lung regeneration: Stem cells and niches

**DOI:** 10.1016/j.mocell.2025.100241

**Published:** 2025-06-13

**Authors:** Hyeyoung Lee, Chae Won Lim, Woo Ram Lee, Ji Eun Park, Da Yeon Yu, Jinwook Choi

**Affiliations:** 1 Department of Life Sciences, Gwangju Institute of Science and Technology, Gwangju 61005, Republic of Korea; 2 Integrated Institute of Biomedical Research, Gwangju Institute of Science and Technology, Gwangju 61005, Republic of Korea

**Keywords:** Alveolar regeneration, Lung organoids, Lung stem cells, Niches, Transitional cell states

## Abstract

The respiratory system is an essential organ that performs gas exchange through the blood circulation in mammals. Unlike other organs, the lungs are directly exposed to the external environment, including particulate matter, cigarette smoke, and various pollutants, and are therefore, highly susceptible to damage. The lungs retain regional-specific stem/progenitor cells that quickly mobilize to replace the damaged epithelium. Accumulating evidences suggest that fate decision of stem cells relies on regulatory programs integrated by niches constituting the microenvironment providing diverse signals that regulate stem cell behavior. Therefore, understanding cellular diversity and precise interaction between stem cells and their respective niches is crucial to understand how tissue recovers homeostasis after injury. Here, in this review, we summarize recent progress in cellular and functional identity of stem cells and distinctive niches in the lungs. We also describe the molecular mechanism of genetic and epigenetic program in the regulation of stem cell behavior during tissue regeneration. Lastly, we introduce the three-dimensional lung organoid platforms that provide valuable insights into the mechanisms of lung pathophysiology in human system.

## INTRODUCTION

The lung is a highly specialized organ that facilitates efficient gas exchange through its vast alveolar surface area and intricate capillary network. Maintaining the structural and functional integrity of this complex tissue is essential for respiratory health, and damage to the alveolar epithelium caused by infections, toxins, or chronic inflammation can result in impaired regeneration and progressive lung diseases such as idiopathic pulmonary fibrosis (IPF) and chronic obstructive pulmonary disease (COPD).

Over the past decade, advances in lineage-tracing, single-cell transcriptomics, and three-dimensional (3D) lung organoid technologies have dramatically expanded our understanding of the cellular and molecular mechanisms that govern lung regeneration. A growing body of evidence suggests that a diverse array of epithelial stem and progenitor cells orchestrate the repair of injured alveolar structures. These epithelial cells do not act in isolation but are influenced by a dynamic microenvironment—or niche—composed of mesenchymal, endothelial, and immune cells that provide essential cues to support regeneration or, conversely, drive pathological remodeling.

In this review, we provide a comprehensive overview of lung epithelial stem/progenitor cell populations involved in alveolar regeneration, with an emphasis on their cellular plasticity and lineage trajectories following injury. We further explore the critical roles of mesenchymal, endothelial, and immune niche cells in regulating epithelial repair. Finally, we highlight the application of 3D organoid models in studying human lung regeneration and modeling disease, offering insights into future therapeutic strategies.

## CELLULAR DIVERSITY AND STEM/PROGENITOR CELLS IN THE LUNG EPITHELIUM

**Fig. 1:**
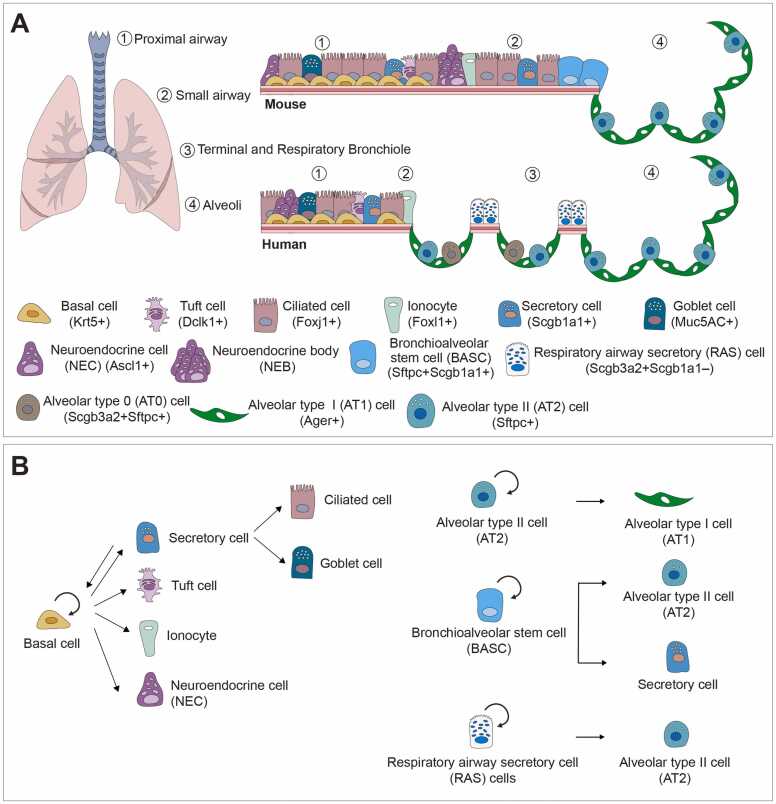
Comparative epithelial cell composition and lineage differentiation potential in mouse and human lungs. (A) Schematic illustration depicting epithelial cell types across 4 anatomical regions—proximal airway, small airway, respiratory airway, and alveoli—in mouse and human lungs. Key differences include the presence of respiratory airway secretory cells (RASCs) and alveolar type 0 (AT0) cells in the distal airways of humans, and bronchioalveolar stem cells (BASCs) at the bronchioalveolar duct junction in mice. (B) Diagram summarizing epithelial lineage hierarchies based on developmental and injury-repair models. In the airways, basal cells serve as progenitors for multiple differentiated cell types. In the alveolar compartment, BASCs (in mice) and RASCs (in humans) give rise to AT2 cells, which further differentiate into alveolar type 1 AT1 cells. These lineage trajectories highlight fundamental species-specific differences in epithelial regeneration and plasticity.

The respiratory tract is a complex system having multiple cell lineages and regional-specific niches alongside proximal-distal axis, which is anatomically divided into 2 major systems of conducting airways and respiratory zone, respectively. Classical studies using histological analysis and recent advanced technologies, including single-cell RNA sequencing, have uncovered a significant degree of cellular diversity and stem/progenitor population within the lungs. In the proximal airways, basal cells characterized by the expression of cytokeratin 5 (KRT5) and transformation-related protein 63 (p63) within the pseudostratified epithelium have been identified as stem cells (Crystal, 2014, Rock et al., 2010). They are capable of differentiating into multilineages of luminal surface of the airways, including secretory and ciliated cells along with more rare cell types, such as tuft cells, ionocytes, and neuroendocrine cells post injury (Montoro et al., 2018, Plasschaert et al., 2018, Surve et al., 2023) (Fig. 1A). In mice, the distal intrapulmonary airways exhibit a relatively uniform structure characterized by the absence of cartilaginous rings and basal stem cells. Their primary role is to conduct air toward the alveoli. These airways are lined with a heterogeneous population of epithelial cells, including both secretory and ciliated cells, along with occasional neuroendocrine cells that cluster into structures of neuroendocrine bodies. Within this region, certain subsets of secretory cells function as local facultative progenitors that contain the ability of self-renewal and differentiating into ciliated cells and goblet cells, and acquire a regenerative potential to dedifferentiate into basal cells in response to injury (Fig. 1B) (Rawlins et al., 2009, Tata et al., 2013). At the bronchioalveolar duct junction—the transition zone where the distal airways of mice connect to the alveolar compartment—a rare population of cells displays multipotency for both airway and alveolar epithelial lineages under certain injury conditions. These cells, known as bronchioalveolar stem cells (BASCs), which are characterized by the coexpression of markers for airway secretory cells (eg, Scgb1a1) and alveolar type 2 (AT2) cells (eg, Sftpc), have potential to give rise to both airway and alveolar lineages upon lung injury (Liu et al., 2019b, Salwig et al., 2019).

In the distal part of pulmonary axis, alveolar epithelium that is a specialized structure for gas exchange is lined by 2 major cell types. Alveolar type 1 (AT1) cells, which cover approximately 95% of the alveolar surface area, have a flat, squamous morphology and are closely associated with microvascular endothelial cells, facilitating gas exchange (Fig. 1A) (Weibel, 2009). AT2 cells are the reservoir of stem cell activity that are able to self-renew and differentiate into AT1 cells during injury repair (Fig. 1B) (Barkauskas et al., 2013). There is growing interest in this region, as damage to the alveoli has been linked to the onset of multiple pulmonary disorders, including emphysema and IPF (Adams et al., 2020, Choi et al., 2020, Habermann et al., 2020, Kobayashi et al., 2020, Strunz et al., 2020, Tsutsumi et al., 2020, Yu et al., 2022).

It is of note that the cellular composition and structural organization of distal airways in larger mammals, including humans, differ notably from those in mice (ten Have-Opbroek et al., 1991). In the human lung, large intrapulmonary airways sequentially branch and constrict into terminal bronchioles, which further develop into respiratory bronchioles (RBs). RBs represent a distinct anatomical structure where alveolar regions are interspersed with airway segments, forming a unique interdigitated architecture (Fig. 1A). Recent studies have identified a specialized population of secretory cells within the RBs of human and ferret lungs, termed respiratory airway secretory (RAS) cells or terminal and respiratory bronchioles secretory cells (TRB-SCs), which possess self-renewal capacity and can differentiate into AT2 cells relying on Notch and Wnt signaling (Basil et al., 2022). Moreover, another subset denoted as alveolar type 0 (AT0) cells expressing both Scgb3a2 and Sftpc was recently uncovered, which represent a transient cell state mediating differentiation of AT2 cells into RASCs/terminal and respiratory bronchioles secretory cells or AT1 cells (Kadur Lakshminarasimha Murthy et al., 2022).

## ALVEOLAR STEM CELLS AND TRANSITIONAL CELL STATES IN ALVEOLAR REGENERATION AND FIBROSIS

Studies leveraging murine models integrating lineage-tracing and lung injury in combination with lung organoids have provided detailed insights into the pathways involved in alveolar regeneration. AT2 cells acquire stem cell properties that are capable of self-renewing and differentiating into AT1 cells, implicating the key population in alveolar regeneration (Barkauskas et al., 2013). Increasing evidences reveal that Axin2+ AT2 cells, distinctive sublineages that respond to Wnt signaling secreted from adjacent fibroblast niches, play a dominant role in alveolar repair process, suggesting a mechanism by which a larger fraction of AT2 cells is recruited for proliferation and differentiation into AT1 cells (Nabhan et al., 2018, Zacharias et al., 2018). In addition, a subset of AT2 cells expressing IL-1R1, a functional receptor for interleukin-1β (IL-1β), selectively proliferates and gives rise to AT1 cells in response to inflammation following injury (Choi et al., 2020). Deletion of IL-1R1 on AT2 cells impairs the differentiation of AT2 cells, resulting in a failure to replenish AT1 cells and affecting alveolar regeneration, highlighting the context-dependent activation of AT2 cells responding to specific signals derived from surrounding niches. While multiple signaling pathways—including the activation of BMP, Notch, Yap/Taz, and TGF-β signaling, as well as the suppression of Wnt signaling—have been implicated in the differentiation of AT2 cells into AT1 cells, the precise mechanisms of AT2 cell differentiation into AT1 cells remained unclear (Al Alam et al., 2011, Chung et al., 2018, Finn et al., 2019, Flozak et al., 2010, Frank et al., 2016, LaCanna et al., 2019, Lange et al., 2015, Liu et al., 2016, Wu et al., 2020).

**Fig. 2:**
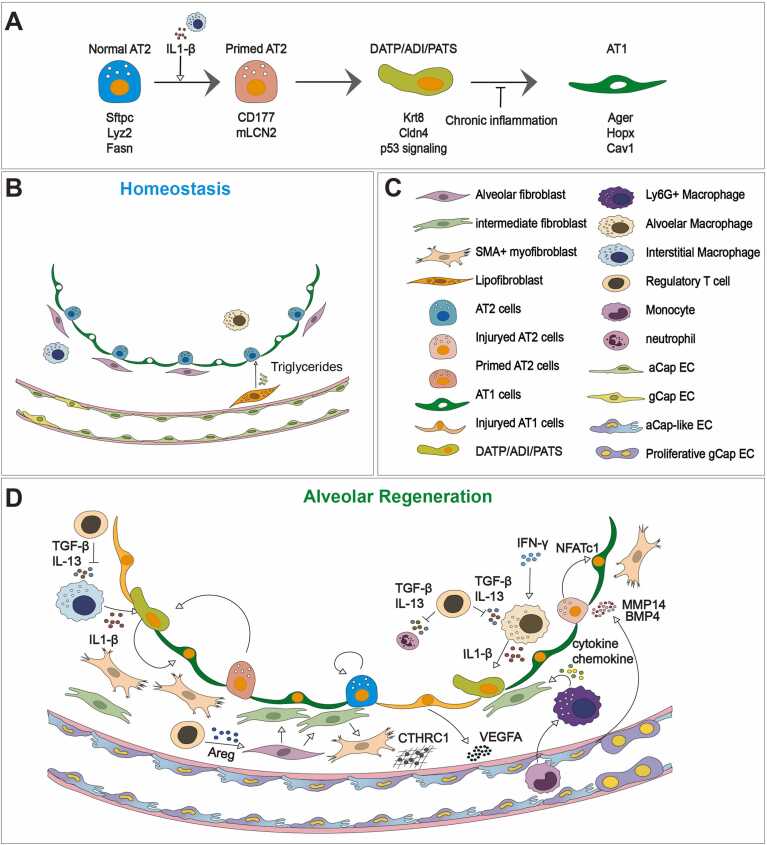
Cellular dynamics of stem cells and their niches during alveolar regeneration. (A) Schematic model of alveolar regeneration. Following injury, IL-1β from interstitial macrophages primes AT2 cells, leading to their transition into intermediate states such as damage-associated transient progenitors (DATPs), pre-alveolar type-1 transitional cells (PATS), or alveolar differentiation intermediates (ADIs). These transitional states are marked by loss of AT2 identity, upregulation of Krt8, Cldn4, and p53 target genes, and a shift toward a squamous morphology. In healthy regeneration, transitional cells resolve into mature AT1 cells. However, under chronic inflammatory conditions—such as pulmonary fibrosis or in lungs of patients with idiopathic pulmonary fibrosis (IPF)—Krt8+ transitional cells persist abnormally, potentially contributing to disease pathogenesis. (B) In the steady-state alveolus, alveolar type 2 (AT2) and type 1 (AT1) cells constitute the epithelial barrier, supported by diverse fibroblast populations and resident immune cells. Lipofibroblasts located beneath AT2 cells contribute to surfactant production through lipid metabolism. Alveolar and interstitial macrophages provide immune surveillance. The underlying vasculature consists of aerocyte (aCap) and general capillary (gCap) endothelial cells forming the alveolar-capillary barrier. (C) Summary of major epithelial, stromal, immune, and endothelial cell populations involved in alveolar homeostasis and regeneration, including transitional and injury-associated states. (D) Following injury, interstitial macrophages and infiltrating monocytes secrete IL-1β, triggering AT2 cells to enter a transitional state known as DATPs. Regulatory T cells (Tregs) contribute to epithelial regeneration through TGF-β and IL-13 signaling. CTHRC1+ myofibroblasts expand and participate in matrix remodeling. Alveolar macrophages, activated by interferon-gamma (IFN-γ), contribute to vascular remodeling. AT1 cells secrete VEGFA to maintain alveolar structure, while endothelial-derived MMP14 and BMP4 promote epithelial regeneration via NFATc1-dependent pathways. Proliferative gCap endothelial cells (ECs) and aCap-like ECs—emerging during injury with partial aCap identity—contribute to vascular reconstruction. Additionally, Ly6G+ macrophages secrete cytokines and chemokines that support tissue repair and inflammation resolution.

Recent studies utilizing single-cell RNA sequencing of lung injury and fibrosis have identified a distinct transitional cell state between AT2 and AT1 cells, referred to as the damage-associated transient progenitors (DATPs), pre-alveolar type-1 transitional cell state (PATS), or alveolar differentiation intermediates (ADIs) (Fig. 2A) (Choi et al., 2020; Kobayashi et al., 2020; Strunz et al., 2020). This transitional population is defined by unique molecular signatures and emerges during the regenerative phase following lung injury. Upon injury, a distinct transitional cell population characterized by the expression of Keratin 8 (KRT8) and Claudin 4 (CLDN4) has been identified, further demonstrated that IL-1β, secreted by interstitial macrophages, can induce a subset of AT2 cells expressing the IL-1 receptor to adopt a DATP phenotype, implicating inflammatory signaling as a key driver of this transitional state (Choi et al., 2020). Similar to PATS and ADI, DATPs show distinctive transcription programs that include activation of DNA damage response pathways (eg, TP53 signaling), cellular senescence, and engagement of transforming growth factor β (TGF-β) signaling (Choi et al., 2020; Kobayashi et al., 2020; Strunz et al., 2020). Disruption of these pathways impairs AT2 differentiation potential, highlighting potential therapeutic targets for attenuating degenerative pulmonary diseases such as lung fibrosis.

Notably, the accumulation of these KRT8^high^ transitional cells (also known as KRT17^+^ basaloid) has also been observed in human lung tissues from patients who succumbed to IPF and COVID-19 or underwent lung transplantation due to post-COVID fibrosis (Adams et al., 2020, Delorey et al., 2021, Dinnon et al., 2022, Habermann et al., 2020, Han et al., 2023, Kobayashi et al., 2020, Melms et al., 2021). Moreover, recent studies highlight that these cell states are also associated with lung tumor development (Choi et al., 2020, England et al., 2025, Han et al., 2024, Hill et al., 2023, Moye et al., 2024). While the accumulation of transitional cells has been consistently associated with lung injury and chronic diseases, their functional role remains under investigation. Whether they are merely markers of ongoing AT2-to-AT1 differentiation or actively contribute to pathology is not yet fully understood. Additionally, it remains unclear whether aging influences the magnitude or persistence of transitional cell populations in response to equivalent injuries.

## STEM CELL NICHES FOR ALVEOLAR REGENERATION

### Fibroblasts in Lung Repair

Fibroblasts are key stromal cells that regulate extracellular matrix (ECM) composition and contribute to tissue homeostasis and repair (Plikus et al., 2021, Talbott et al., 2022). In the lungs, 4 different types of population have been identified in homeostasis based on anatomical localization and molecular signatures, including alveolar fibroblasts (Wnt2+, Plin2+, and Ces1d+), peribronchial fibroblasts (Lgr6+, Hhip+), adventitial fibroblasts (Pi16+, Dcn+), and myofibroblasts (Acta2+, Myh11+) (Tsukui et al., 2020, Tsukui et al., 2024, Zepp et al., 2017). Alveolar fibroblasts play essential roles in supplying triglycerides required for surfactant production by AT2 cells (Fig. 2B-D) (Taghizadeh et al., 2021). Upon injury, fibroblasts transition through intermediate states, acquiring inflammatory or fibrotic phenotypes, or even tumor-associated characteristics (Zhang and Liu, 2024). During alveolar damage, PDGFα+ fibroblasts support euplastic repair via Wnt and FGF signaling, whereas severe injury (eg, influenza) shifts them to a PDGFβ+ state, promoting KRT5+ basal cell–mediated dysplastic repair via Notch signaling (Jones et al., 2024). In acute respiratory distress syndrome, fibroblast-derived cytokines such as ADAMTS4 enhance immune cell infiltration, exacerbating lung dysfunction (Boyd et al., 2020). Recently, a novel subset of fibroblasts emerging during fibrotic environment have been identified. Fibrotic lungs exhibit expansion of CTHRC1+ fibroblasts, which upregulate ECM production in response to TGF-β from immune cells (Tsukui et al., 2020, Tsukui et al., 2024). TGF-β also suppresses SFRP1 in lipofibroblasts, promoting their conversion to profibrotic myofibroblasts (Konkimalla et al., 2023). Aberrant fibroblast states influence not only ECM remodeling but also alveolar epithelial stem cell dynamics, highlighting their therapeutic relevance in lung injury and repair. Therefore, investigating mechanisms underlying these fibroblast transitions—as well as their signaling pathways—could pave the way for developing therapies promoting euplastic repair while preventing fibrosis during conditions like lung injury or viral infections.

### Vascular Niche in Lung Regeneration

The lung is a gas exchange organ supported by a dense vascular network essential for efficient gas exchange, which occurs across the alveolar-capillary barrier, a thin interface comprising epithelial, mesenchymal, and endothelial cells (ECs). Recent studies identified 2 distinctive subsets of capillary ECs: aerocyte cells (aCap) marked by carbonic anhydrase 4 (Car4), and general capillary cells (Gillich et al., 2020, Vila Ellis et al., 2020). Following injury, aCap specialized for gas exchange and leukocyte trafficking play an essential role by generating new vasculature from pre-existing blood vessels in areas lacking vascular networks and by promoting the formation of new alveoli, thereby contributing to the restoration of lung function (Fig. 2B-D) (Niethamer et al., 2020, Potente et al., 2011, Rafii et al., 2016). Vascular endothelial growth factor A (VEGFA) secreted by AT1 cells activates Car4⁺ ECs via VEGFR2, preserving alveolar architecture (Ellis et al., 2020, Niethamer et al., 2020, Yang et al., 2016). In addition, EC-derived MMP14 and BMP4 further promote epithelial progenitor cell expansion and BASC differentiation, respectively, via EGF-like ligands and NFATc1-dependent pathways (Ding et al., 2011, Lee et al., 2014). In summary, ECs orchestrate vascular and epithelial regeneration through angiogenesis, trophic signaling, and cellular cross-talk, playing a central role in restoring lung function.

### Immune Niche in Lung Regeneration

As a primary interface with the external environment, the lungs are continuously exposed to pathogens and toxic substances, making them vulnerable to injury. To counter these threats, they are equipped with both innate and adaptive immune cells that initiate inflammatory responses through cytokine release (Kawasaki et al., 2022, Kumar, 2020, Xu et al., 2023). However, excessive or uncontrolled inflammation impairs lung regeneration, promoting chronic lung diseases such as IPF and COPD (Choi et al., 2020, Eming et al., 2017). Significantly, macrophages and regulatory T cells (Tregs) are particularly involved in promoting epithelial regeneration (Fig. 2B-D). Lung macrophages are classified into alveolar macrophages derived from yolk sac during development and interstitial macrophages replenishing from circulating monocytes (Bain and MacDonald, 2022, Liegeois et al., 2018, Murray and Wynn, 2011). Recent research has revealed that during SARS-CoV-2 infection, an increase in interferon-gamma levels suppresses peroxisome biogenesis in alveolar macrophages while promoting pexophagy. This dysfunction of peroxisomes leads to mitochondrial damage, inflammasome activation, and increased secretion of IL-1β (Wei et al., 2025). Additionally, IL-1β secreted by interstitial macrophages has been shown to drive AT2 cells to transition into KRT8+ cells, suggesting a potential link to postacute COVID-19 sequelae (Choi et al., 2020). Furthermore, Ly6G+ macrophages, derived from monocytes and located near regenerating AT2 cells, secrete cytokines (TNF-α, IL-10, and IL-1α) and chemokines (CCL5, CXCL16, CCL12, and CXCL10) via IL-4R signaling, supporting epithelial regeneration (Ruscitti et al., 2024). Tregs facilitate tissue regeneration through multiple mechanisms. During acute lung injury, they induce neutrophil apoptosis via TGF-β (D'Alessio et al., 2009, Lewkowicz et al., 2006). Damaged epithelial cells release IL-33, which binds to ST2 on Tregs, triggering secretion of amphiregulin and IL-13 (Kaiser et al., 2023, Liu et al., 2019a, Zhou et al., 2023). Amphiregulin binds to EGFR on mesenchymal cells, stimulating the production of FGF7 and FGF10, which enhance AT2 cell proliferation and differentiation (Kaiser et al., 2023). IL-13 also limits neutrophil and macrophage infiltration and enhances macrophage efferocytosis via the IL-13-IL-10-VAV1-RAC1 axis, promoting inflammation resolution and tissue repair (Proto et al., 2018). Further studies are warranted to elucidate how these immune cells coordinate repair processes by remodeling alveolar niches and how they can be targeted for therapeutic strategy.

## LUNG ORGANOIDS FOR HUMAN PATHOPHYSIOLOGY, DISEASE MODELING, AND DRUG DEVELOPMENT

**Fig. 3:**
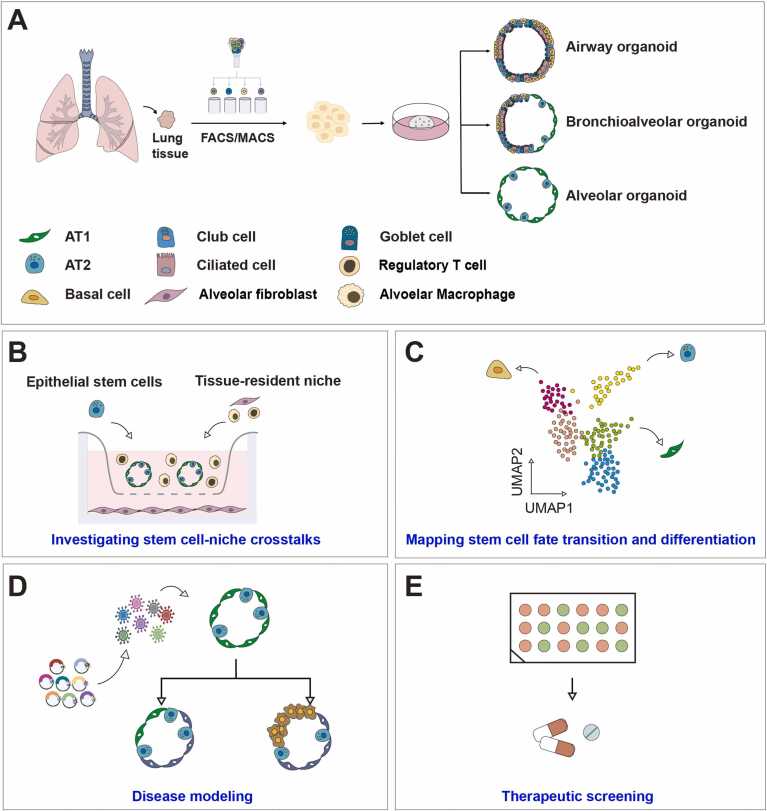
Schematic overview of human lung organoid establishment and applications. (A) Adult human lung tissue is enzymatically dissociated to obtain a single-cell suspension. Epithelial stem/progenitor cells are subsequently isolated using fluorescence-activated (FACS) or magnetic-activated (MACS) cell sorting. These cells are embedded in extracellular matrix–based hydrogels and cultured under defined conditions to generate lung organoids. Depending on the cellular origin and culture conditions, the resulting organoids can differentiate into airway, bronchioalveolar, or alveolar subtypes. (B) Coculture systems allow investigation of interactions between epithelial cells and their surrounding niche, including stromal or immune cells, facilitating the study of cell-cell communication and microenvironmental influence. (C) Organoids serve as a model to explore lineage commitment and differentiation trajectories of lung stem and progenitor cells under homeostatic or injury-mimicking conditions. (D) Patient-derived lung organoids retain clinically relevant cellular and molecular characteristics, enabling disease modeling of genetic and acquired pulmonary disorders. (E) Organoids provide a physiologically relevant platform for preclinical drug screening and evaluation of therapeutic efficacy.

Organoids are 3-dimensional cell culture systems derived from tissue-specific stem cells isolated from adult human organs, typically grown in ECM components or hydrogels containing collagen (Barkauskas et al., 2017). Under defined conditions, these cells proliferate, differentiate, and self-organize to form epithelial structures resembling native lung architecture (Pauli et al., 2017, Sasai et al., 2012, Shamir and Ewald, 2014) (Fig. 3A). Organoids from tracheobronchial and alveolar tissues successfully mimic epithelial organization in vitro (Barkauskas et al., 2013; Dye et al., 2015), and human-derived systems are particularly valuable for modeling diseases not fully captured by murine models (Nikolic and Rawlins, 2017).

To better simulate in vivo conditions, coculture systems have been developed (Fig. 3B-E). Alveolar organoids with fibroblasts reproduce epithelial-stromal interactions characteristic of fibrotic lungs and are being used for drug testing in IPF (Suezawa et al., 2021). In oncology, 3D coculture organoids derived from cancer patients allow the study of tumor-fibroblast interactions, immune responses, and prediction of drug-responsiveness (Balazova et al., 2023). Lung organoids also serve as models for infectious and chronic diseases. For example, RSV-infected airway organoids help elucidate epithelial remodeling and immune recruitment (Sachs et al., 2019). Lung organoids have also been applied to disease modeling for understanding mechanisms underlying lung disease initiation and progression such as COPD, asthma, cystic fibrosis, and IPF (Chen et al., 2017, Danahay et al., 2015, Jacob et al., 2017, Kobayashi et al., 2020, McCauley et al., 2017). In summary, lung organoids provide a robust ex vivo model for studying regeneration, disease pathogenesis, and therapeutic screening, owing to their ability to replicate native tissue structure and cellular interactions.

## DISCUSSION

The lung is a highly complex organ composed of diverse cell types that orchestrate its essential functions in gas exchange, host defense, and regeneration. Over the past decade, advances in single-cell technologies and in vitro organoid models have dramatically expanded our understanding of lung epithelial stem/progenitor cell populations and their interactions with specialized niches, including stromal, vascular, and immune components. These studies have revealed that regeneration following injury is not a uniform process but instead involves dynamic and context-specific cellular programs. AT2 cells and their transitional intermediates play central roles in alveolar repair, while interactions with fibroblasts, endothelial cells, and immune populations tightly regulate this process through paracrine signaling and structural support.

Despite significant progress, several critical questions remain. The functional significance of transitional states such as DATPs and their persistence in chronic lung diseases like fibrosis or post viral sequelae is not yet fully understood. Similarly, how aging alters niche-stem cell interactions and regenerative capacity requires further exploration. The discovery of new fibroblast subsets, endothelial heterogeneity, and immune cell functions in regeneration suggests that targeting specific niche components could offer novel therapeutic avenues to promote repair or prevent pathological remodeling.

Emerging 3D organoid models provide an invaluable platform to interrogate human-specific mechanisms of lung development, disease pathogenesis, and response to therapies. These systems offer the opportunity to bridge the translational gap between mouse models and human lung biology. Incorporating immune and mesenchymal compartments into organoid cultures will be key to capturing the full complexity of the lung niche. As the field moves forward, integrating multiomics, spatial transcriptomics, and high-content imaging with these models will further refine our understanding of lung regeneration and open new possibilities for precision medicine in respiratory diseases.

## Author Contributions

**Chae Won Lim:** Writing – review & editing, Writing – original draft. **Woo Ram Lee:** Writing – review & editing, Writing – original draft. **Jinwook Choi:** Writing – review & editing, Writing – original draft, Funding acquisition, Conceptualization. **Hyeyoung Lee:** Writing – review & editing, Writing – original draft. **Ji Eun Park:** Writing – review & editing, Writing – original draft. **Da Yeon Yu:** Writing – review & editing, Writing – original draft.

## Declaration of Competing Interests

The authors have no potential conflicts of interest to disclose.

## References

[bib1] Adams T.S., Schupp J.C., Poli S., Ayaub E.A., Neumark N., Ahangari F., Chu S.G., Raby B.A., DeIuliis G., Januszyk M. (2020). Single-cell RNA-seq reveals ectopic and aberrant lung-resident cell populations in idiopathic pulmonary fibrosis. Sci. Adv..

[bib2] Al Alam D., Green M., Tabatabai Irani R., Parsa S., Danopoulos S., Sala F.G., Branch J., El Agha E., Tiozzo C., Voswinckel R. (2011). Contrasting expression of canonical Wnt signaling reporters TOPGAL, BATGAL and Axin2(LacZ) during murine lung development and repair. PLoS One.

[bib3] Bain C.C., MacDonald A.S. (2022). The impact of the lung environment on macrophage development, activation and function: diversity in the face of adversity. Mucosal. Immunol..

[bib4] Balazova K., Clevers H., Dost A.F.M. (2023). The role of macrophages in non-small cell lung cancer and advancements in 3D co-cultures. Elife.

[bib5] Barkauskas C.E., Chung M.I., Fioret B., Gao X., Katsura H., Hogan B.L. (2017). Lung organoids: current uses and future promise. Development.

[bib6] Barkauskas C.E., Cronce M.J., Rackley C.R., Bowie E.J., Keene D.R., Stripp B.R., Randell S.H., Noble P.W., Hogan B.L. (2013). Type 2 alveolar cells are stem cells in adult lung. J. Clin. Invest..

[bib7] Basil M.C., Cardenas-Diaz F.L., Kathiriya J.J., Morley M.P., Carl J., Brumwell A.N., Katzen J., Slovik K.J., Babu A., Zhou S. (2022). Human distal airways contain a multipotent secretory cell that can regenerate alveoli. Nature.

[bib8] Boyd D.F., Allen E.K., Randolph A.G., Guo X.J., Weng Y., Sanders C.J., Bajracharya R., Lee N.K., Guy C.S., Vogel P. (2020). Exuberant fibroblast activity compromises lung function via ADAMTS4. Nature.

[bib9] Chen Y.W., Huang S.X., de Carvalho A., Ho S.H., Islam M.N., Volpi S., Notarangelo L.D., Ciancanelli M., Casanova J.L., Bhattacharya J. (2017). A three-dimensional model of human lung development and disease from pluripotent stem cells. Nat. Cell Biol..

[bib10] Choi J., Park J.E., Tsagkogeorga G., Yanagita M., Koo B.K., Han N., Lee J.H. (2020). Inflammatory signals induce AT2 cell-derived damage-associated transient progenitors that mediate alveolar regeneration. Cell Stem Cell.

[bib11] Chung M.I., Bujnis M., Barkauskas C.E., Kobayashi Y., Hogan B.L.M. (2018). Niche-mediated BMP/SMAD signaling regulates lung alveolar stem cell proliferation and differentiation. Development.

[bib12] Crystal R.G. (2014). Airway basal cells the “smoking gun” of chronic obstructive pulmonary disease. Am. J. Resp. Crit. Care.

[bib13] D'Alessio F.R., Tsushima K., Aggarwal N.R., West E.E., Willett M.H., Britos M.F., Pipeling M.R., Brower R.G., Tuder R.M., McDyer J.F. (2009). CD4+CD25+Foxp3+ Tregs resolve experimental lung injury in mice and are present in humans with acute lung injury. J. Clin. Invest..

[bib14] Danahay H., Pessotti A.D., Coote J., Montgomery B.E., Xia D., Wilson A., Yang H., Wang Z., Bevan L., Thomas C. (2015). Notch2 is required for inflammatory cytokine-driven goblet cell metaplasia in the lung. Cell Rep..

[bib15] Delorey T.M., Ziegler C.G.K., Heimberg G., Normand R., Yang Y., Segerstolpe A., Abbondanza D., Fleming S.J., Subramanian A., Montoro D.T. (2021). COVID-19 tissue atlases reveal SARS-CoV-2 pathology and cellular targets. Nature.

[bib16] Ding B.-S., Nolan D.J., Guo P., Babazadeh A.O., Cao Z., Rosenwaks Z., Crystal R.G., Simons M., Sato T.N., Worgall S. (2011). Endothelial-derived angiocrine signals induce and sustain regenerative lung alveolarization. Cell.

[bib17] Dinnon K.H., 3rd, Leist S.R., Okuda K., Dang H., Fritch E.J., Gully K.L., De la Cruz G., Evangelista M.D., Asakura T., Gilmore R.C. (2022). SARS-CoV-2 infection produces chronic pulmonary epithelial and immune cell dysfunction with fibrosis in mice. Sci. Transl. Med..

[bib18] Dye B.R., Hill D.R., Ferguson M.A., Tsai Y.H., Nagy M.S., Dyal R., Wells J.M., Mayhew C.N., Nattiv R., Klein O.D. (2015). In vitro generation of human pluripotent stem cell derived lung organoids. Elife.

[bib19] Ellis L.V., Cain M.P., Hutchison V., Flodby P., Crandall E.D., Borok Z., Zhou B., Ostrin E.J., Wythe J.D., Chen J. (2020). Epithelial Vegfa specifies a distinct endothelial population in the mouse lung. Dev. Cell.

[bib20] Eming S.A., Wynn T.A., Martin P. (2017). Inflammation and metabolism in tissue repair and regeneration. Science.

[bib21] England F.J., Bordeu I., Ng M.E., Bang J., Kim B., Choi J., Cardoso E.C., Koo B.K., Simons B.D., Lee J.H. (2025). Sustained NF-kappaB activation allows mutant alveolar stem cells to co-opt a regeneration program for tumor initiation. Cell Stem Cell.

[bib22] Finn J., Sottoriva K., Pajcini K.V., Kitajewski J.K., Chen C., Zhang W., Malik A.B., Liu Y. (2019). Dlk1-mediated temporal regulation of notch signaling is required for differentiation of alveolar type II to type I cells during repair. Cell Rep..

[bib23] Flozak A.S., Lam A.P., Russell S., Jain M., Peled O.N., Sheppard K.A., Beri R., Mutlu G.M., Budinger G.R., Gottardi C.J. (2010). Beta-catenin/T-cell factor signaling is activated during lung injury and promotes the survival and migration of alveolar epithelial cells. J. Biol. Chem..

[bib24] Frank D.B., Peng T., Zepp J.A., Snitow M., Vincent T.L., Penkala I.J., Cui Z., Herriges M.J., Morley M.P., Zhou S. (2016). Emergence of a wave of Wnt signaling that regulates lung alveologenesis by controlling epithelial self-renewal and differentiation. Cell Rep..

[bib25] Gillich A., Zhang F., Farmer C.G., Travaglini K.J., Tan S.Y., Gu M., Zhou B., Feinstein J.A., Krasnow M.A., Metzger R.J. (2020). Capillary cell-type specialization in the alveolus. Nature.

[bib26] Habermann A.C., Gutierrez A.J., Bui L.T., Yahn S.L., Winters N.I., Calvi C.L., Peter L., Chung M.I., Taylor C.J., Jetter C. (2020). Single-cell RNA sequencing reveals profibrotic roles of distinct epithelial and mesenchymal lineages in pulmonary fibrosis. Sci. Adv..

[bib27] Han G., Sinjab A., Rahal Z., Lynch A.M., Treekitkarnmongkol W., Liu Y., Serrano A.G., Feng J., Liang K., Khan K. (2024). An atlas of epithelial cell states and plasticity in lung adenocarcinoma. Nature.

[bib28] Han S., Lee M., Shin Y., Giovanni R., Chakrabarty R.P., Herrerias M.M., Dada L.A., Flozak A.S., Reyfman P.A., Khuder B. (2023). Mitochondrial integrated stress response controls lung epithelial cell fate. Nature.

[bib29] Hill W., Lim E.L., Weeden C.E., Lee C., Augustine M., Chen K., Kuan F.C., Marongiu F., Evans E.J., Moore D.A. (2023). Lung adenocarcinoma promotion by air pollutants. Nature.

[bib30] Jacob A., Morley M., Hawkins F., McCauley K.B., Jean J.C., Heins H., Na C.L., Weaver T.E., Vedaie M., Hurley K. (2017). Differentiation of human pluripotent stem cells into functional lung alveolar epithelial cells. Cell Stem Cell.

[bib31] Jones D.L., Morley M.P., Li X., Ying Y., Zhao G., Schaefer S.E., Rodriguez L.R., Cardenas-Diaz F.L., Li S., Zhou S. (2024). An injury-induced mesenchymal-epithelial cell niche coordinates regenerative responses in the lung. Science.

[bib32] Kadur Lakshminarasimha Murthy P., Sontake V., Tata A., Kobayashi Y., Macadlo L., Okuda K., Conchola A.S., Nakano S., Gregory S., Miller L.A. (2022). Human distal lung maps and lineage hierarchies reveal a bipotent progenitor. Nature.

[bib33] Kaiser K.A., Loffredo L.F., Santos-Alexis K.L., Ringham O.R., Arpaia N. (2023). Regulation of the alveolar regenerative niche by amphiregulin-producing regulatory T cells. J. Exp. Med..

[bib34] Kawasaki T., Ikegawa M., Yunoki K., Otani H., Ori D., Ishii K.J., Kuroda E., Takamura S., Kitabatake M., Ito T. (2022). Alveolar macrophages instruct CD8(+) T cell expansion by antigen cross-presentation in lung. Cell Rep..

[bib35] Kobayashi Y., Tata A., Konkimalla A., Katsura H., Lee R.F., Ou J., Banovich N.E., Kropski J.A., Tata P.R. (2020). Persistence of a regeneration-associated, transitional alveolar epithelial cell state in pulmonary fibrosis. Nat. Cell Biol..

[bib36] Konkimalla A., Konishi S., Macadlo L., Kobayashi Y., Farino Z.J., Miyashita N., El Haddad L., Morowitz J., Barkauskas C.E., Agarwal P. (2023). Transitional cell states sculpt tissue topology during lung regeneration. Cell Stem Cell.

[bib37] Kumar V. (2020). Pulmonary innate immune response determines the outcome of inflammation during pneumonia and sepsis-associated acute lung injury. Front. Immunol..

[bib38] LaCanna R., Liccardo D., Zhang P., Tragesser L., Wang Y., Cao T., Chapman H.A., Morrisey E.E., Shen H., Koch W.J. (2019). Yap/Taz regulate alveolar regeneration and resolution of lung inflammation. J. Clin. Invest..

[bib39] Lange A.W., Sridharan A., Xu Y., Stripp B.R., Perl A.K., Whitsett J.A. (2015). Hippo/Yap signaling controls epithelial progenitor cell proliferation and differentiation in the embryonic and adult lung. J. Mol. Cell Biol..

[bib40] Lee J.H., Bhang D.H., Beede A., Huang T.L., Stripp B.R., Bloch K.D., Wagers A.J., Tseng Y.H., Ryeom S., Kim C.F. (2014). Lung stem cell differentiation in mice directed by endothelial cells via a BMP4-NFATc1-thrombospondin-1 axis. Cell.

[bib41] Lewkowicz P., Lewkowicz N., Sasiak A., Tchorzewski H. (2006). Lipopolysaccharide-activated CD4+CD25+ T regulatory cells inhibit neutrophil function and promote their apoptosis and death. J. Immunol..

[bib42] Liegeois M., Legrand C., Desmet C.J., Marichal T., Bureau F. (2018). The interstitial macrophage: a long-neglected piece in the puzzle of lung immunity. Cell Immunol..

[bib43] Liu Q., Dwyer G.K., Zhao Y., Li H., Mathews L.R., Chakka A.B., Chandran U.R., Demetris J.A., Alcorn J.F., Robinson K.M. (2019). IL-33-mediated IL-13 secretion by ST2+ Tregs controls inflammation after lung injury. JCI Insight.

[bib44] Liu Q., Liu K., Cui G., Huang X., Yao S., Guo W., Qin Z., Li Y., Yang R., Pu W. (2019). Lung regeneration by multipotent stem cells residing at the bronchioalveolar-duct junction. Nat. Genet..

[bib45] Liu Z., Wu H., Jiang K., Wang Y., Zhang W., Chu Q., Li J., Huang H., Cai T., Ji H. (2016). MAPK-mediated YAP activation controls mechanical-tension-induced pulmonary alveolar regeneration. Cell Rep..

[bib46] McCauley K.B., Hawkins F., Serra M., Thomas D.C., Jacob A., Kotton D.N. (2017). Efficient derivation of functional human airway epithelium from pluripotent stem cells via temporal regulation of Wnt signaling. Cell Stem Cell.

[bib47] Melms J.C., Biermann J., Huang H., Wang Y., Nair A., Tagore S., Katsyv I., Rendeiro A.F., Amin A.D., Schapiro D. (2021). A molecular single-cell lung atlas of lethal COVID-19. Nature.

[bib48] Montoro D.T., Haber A.L., Biton M., Vinarsky V., Lin B., Birket S.E., Yuan F., Chen S., Leung H.M., Villoria J. (2018). A revised airway epithelial hierarchy includes CFTR-expressing ionocytes. Nature.

[bib49] Moye A.L., Dost A.F., Ietswaart R., Sengupta S., Ya V., Aluya C., Fahey C.G., Louie S.M., Paschini M., Kim C.F. (2024). Early-stage lung cancer is driven by a transitional cell state dependent on a KRAS-ITGA3-SRC axis. EMBO J..

[bib50] Murray P.J., Wynn T.A. (2011). Protective and pathogenic functions of macrophage subsets. Nat. Rev. Immunol..

[bib51] Nabhan A.N., Brownfield D.G., Harbury P.B., Krasnow M.A., Desai T.J. (2018). Single-cell Wnt signaling niches maintain stemness of alveolar type 2 cells. Science.

[bib52] Niethamer T.K., Stabler C.T., Leach J.P., Zepp J.A., Morley M.P., Babu A., Zhou S., Morrisey E.E. (2020). Defining the role of pulmonary endothelial cell heterogeneity in the response to acute lung injury. eLife.

[bib53] Nikolic M.Z., Rawlins E.L. (2017). Lung organoids and their use to study cell-cell interaction. Curr. Pathobiol. Rep..

[bib54] Pauli C., Hopkins B.D., Prandi D., Shaw R., Fedrizzi T., Sboner A., Sailer V., Augello M., Puca L., Rosati R. (2017). Personalized in vitro and in vivo cancer models to guide precision medicine. Cancer Discov..

[bib55] Plasschaert L.W., Zilionis R., Choo-Wing R., Savova V., Knehr J., Roma G., Klein A.M., Jaffe A.B. (2018). A single-cell atlas of the airway epithelium reveals the CFTR-rich pulmonary ionocyte. Nature.

[bib56] Plikus M.V., Wang X., Sinha S., Forte E., Thompson S.M., Herzog E.L., Driskell R.R., Rosenthal N., Biernaskie J., Horsley V. (2021). Fibroblasts: origins, definitions, and functions in health and disease. Cell.

[bib57] Potente M., Gerhardt H., Carmeliet P. (2011). Basic and therapeutic aspects of angiogenesis. Cell.

[bib58] Proto J.D., Doran A.C., Gusarova G., Yurdagul A., Sozen E., Subramanian M., Islam M.N., Rymond C.C., Du J., Hook J. (2018). Regulatory T cells promote macrophage efferocytosis during inflammation resolution. Immunity.

[bib59] Rafii S., Butler J.M., Ding B.-S. (2016). Angiocrine functions of organ-specific endothelial cells. Nature.

[bib60] Rawlins E.L., Okubo T., Xue Y., Brass D.M., Auten R.L., Hasegawa H., Wang F., Hogan B.L. (2009). The role of Scgb1a1+ Clara cells in the long-term maintenance and repair of lung airway, but not alveolar, epithelium. Cell Stem Cell.

[bib61] Rock J.R., Randell S.H., Hogan B.L. (2010). Airway basal stem cells: a perspective on their roles in epithelial homeostasis and remodeling. Dis. Model Mech..

[bib62] Ruscitti C., Abinet J., Marechal P., Meunier M., de Meeus C., Vanneste D., Janssen P., Dourcy M., Thiry M., Bureau F. (2024). Recruited atypical Ly6G(+) macrophages license alveolar regeneration after lung injury. Sci. Immunol..

[bib63] Sachs N., Papaspyropoulos A., Zomer-van Ommen D.D., Heo I., Bottinger L., Klay D., Weeber F., Huelsz-Prince G., Iakobachvili N., Amatngalim G.D. (2019). Long-term expanding human airway organoids for disease modeling. EMBO J..

[bib64] Salwig I., Spitznagel B., Vazquez-Armendariz A.I., Khalooghi K., Guenther S., Herold S., Szibor M., Braun T. (2019). Bronchioalveolar stem cells are a main source for regeneration of distal lung epithelia in vivo. EMBO J..

[bib65] Sasai Y., Eiraku M., Suga H. (2012). In vitro organogenesis in three dimensions: self-organising stem cells. Development.

[bib66] Shamir E.R., Ewald A.J. (2014). Three-dimensional organotypic culture: experimental models of mammalian biology and disease. Nat. Rev. Mol. Cell Biol..

[bib67] Strunz M., Simon L.M., Ansari M., Kathiriya J.J., Angelidis I., Mayr C.H., Tsidiridis G., Lange M., Mattner L.F., Yee M. (2020). Alveolar regeneration through a Krt8+ transitional stem cell state that persists in human lung fibrosis. Nat. Commun..

[bib68] Suezawa T., Kanagaki S., Moriguchi K., Masui A., Nakao K., Toyomoto M., Tamai K., Mikawa R., Hirai T., Murakami K. (2021). Disease modeling of pulmonary fibrosis using human pluripotent stem cell-derived alveolar organoids. Stem Cell Rep..

[bib69] Surve M.V., Lin B., Reedy J.L., Crossen A.J., Xu A., Klein B.S., Vyas J.M., Rajagopal J. (2023). Single-cell transcriptomes, lineage, and differentiation of functional airway microfold cells. Am. J. Respir. Cell Mol. Biol..

[bib70] Taghizadeh S., Heiner M., Vazquez-Armendariz A.I., Wilhelm J., Herold S., Chen C., Zhang J.S., Bellusci S. (2021). Characterization in mice of the resident mesenchymal niche maintaining AT2 stem cell proliferation in homeostasis and disease. Stem Cells.

[bib71] Talbott H.E., Mascharak S., Griffin M., Wan D.C., Longaker M.T. (2022). Wound healing, fibroblast heterogeneity, and fibrosis. Cell Stem Cell.

[bib72] Tata P.R., Mou H., Pardo-Saganta A., Zhao R., Prabhu M., Law B.M., Vinarsky V., Cho J.L., Breton S., Sahay A. (2013). Dedifferentiation of committed epithelial cells into stem cells in vivo. Nature.

[bib73] ten Have-Opbroek A.A., Otto-Verberne C.J., Dubbeldam J.A., Dykman J.H. (1991). The proximal border of the human respiratory unit, as shown by scanning and transmission electron microscopy and light microscopical cytochemistry. Anat. Rec..

[bib74] Tsukui T., Sun K.H., Wetter J.B., Wilson-Kanamori J.R., Hazelwood L.A., Henderson N.C., Adams T.S., Schupp J.C., Poli S.D., Rosas I.O. (2020). Collagen-producing lung cell atlas identifies multiple subsets with distinct localization and relevance to fibrosis. Nat. Commun..

[bib75] Tsukui T., Wolters P.J., Sheppard D. (2024). Alveolar fibroblast lineage orchestrates lung inflammation and fibrosis. Nature.

[bib76] Tsutsumi A., Ozaki M., Chubachi S., Irie H., Sato M., Kameyama N., Sasaki M., Ishii M., Hegab A.E., Betsuyaku T. (2020). Exposure to cigarette smoke enhances the stemness of alveolar type 2 cells. Am. J. Respir. Cell Mol. Biol..

[bib77] Vila Ellis L., Cain M.P., Hutchison V., Flodby P., Crandall E.D., Borok Z., Zhou B., Ostrin E.J., Wythe J.D., Chen J. (2020). Epithelial vegfa specifies a distinct endothelial population in the mouse lung. Dev. Cell..

[bib78] Wei X., Qian W., Narasimhan H., Chan T., Liu X., Arish M., Young S., Li C., Cheon I.S., Yu Q. (2025). Macrophage peroxisomes guide alveolar regeneration and limit SARS-CoV-2 tissue sequelae. Science.

[bib79] Weibel E.R. (2009). What makes a good lung?. Swiss Med. Wkly..

[bib80] Wu H., Yu Y., Huang H., Hu Y., Fu S., Wang Z., Shi M., Zhao X., Yuan J., Li J. (2020). Progressive pulmonary fibrosis is caused by elevated mechanical tension on alveolar stem cells. Cell.

[bib81] Xu Y., Lan P., Wang T. (2023). The role of immune cells in the pathogenesis of idiopathic pulmonary fibrosis. Medicina (Kaunas).

[bib82] Yang J., Hernandez B.J., Martinez Alanis D., Narvaez del Pilar O., Vila-Ellis L., Akiyama H., Evans S.E., Ostrin E.J., Chen J. (2016). The development and plasticity of alveolar type 1 cells. Development.

[bib83] Yu H., Lin Y., Zhong Y., Guo X., Lin Y., Yang S., Liu J., Xie X., Sun Y., Wang D. (2022). Impaired AT2 to AT1 cell transition in PM2.5-induced mouse model of chronic obstructive pulmonary disease. Respir. Res..

[bib84] Zacharias W.J., Frank D.B., Zepp J.A., Morley M.P., Alkhaleel F.A., Kong J., Zhou S., Cantu E., Morrisey E.E. (2018). Regeneration of the lung alveolus by an evolutionarily conserved epithelial progenitor. Nature.

[bib85] Zepp J.A., Zacharias W.J., Frank D.B., Cavanaugh C.A., Zhou S., Morley M.P., Morrisey E.E. (2017). Distinct mesenchymal lineages and niches promote epithelial self-renewal and myofibrogenesis in the lung. Cell.

[bib86] Zhang J., Liu Y. (2024). Epithelial stem cells and niches in lung alveolar regeneration and diseases. Chin. Med. J. Pulm. Crit. Care Med..

[bib87] Zhou Y., Xu Z., Liu Z. (2023). Role of IL-33-ST2 pathway in regulating inflammation: current evidence and future perspectives. J. Transl. Med..

